# Supplementary material for: Hypoactive Visual Cortex, Prefrontal Cortex and Insula during Self-Face Recognition in Adults with First-Episode Major Depressive Disorder
Source: Biomedicines. 2023 Aug 4;11(8):2200. doi: 10.3390/biomedicines11082200 (PMC10452386; doi:10.3390/biomedicines11082200)
Supplement: Supplementary file 1 [file biomedicines-11-02200-s001.zip › biomedicines-2509026-supplementary.pdf]

biomedicines

MDPI

Supplementary Materials

Hypoactive Visual Cortex, Prefrontal Cortex and Insula during Self-Face Recognition in Adults with First-Episode Major Depressive Disorder

Table S1. Medication information of adult patients with FEMDD.

Medication information	Number of cases	Duration of medication (days)
Not taking any medication	44	0
Taking venlafaxine alone	3	14,14,7
Taking venlafaxine/ mirtazapine/ oxazepam	2	9,10
Taking venlafaxine/ mirtazapine	2	4,14
Taking venlafaxine/ oxazepam	1	14
Taking venlafaxine/ oxazepam/ zolpidem	1	7
Taking escitalopram alone	4	13,8,7,3
Taking escitalopram/ trazodone	1	11
Taking escitalopram/ oxazepam	1	14

Notes: 9 patients were taking venlafaxine 75-150mg/day and 6 patients were taking escitalopram 5-10mg/day. Among them, 8 patients received a combination of oxazepam 7.5-15mg/day or zolpidem tartrate 5-10mg/day or trazodone 25-50mg/day or mirtazapine 7.5-15mg/day and had been instructed to discontinue the medication 24 hours before the scan. Abbreviations: FEMDD, first-episode major depressive disorder.

The images of faces used in the present study (Figure1).

Thirty-six photos of facial expressions were selected from the Chinese facial affective picture system, among which included 12 male photos with neutral facial expressions (A1-A12), 12 female photos with neutral facial expressions (C1-C12), and 12 photos of other strangers' disgust face (D1-D12), one photo of each participant's self-neutral face (picture S) and one black background picture (BCP) (picture B).

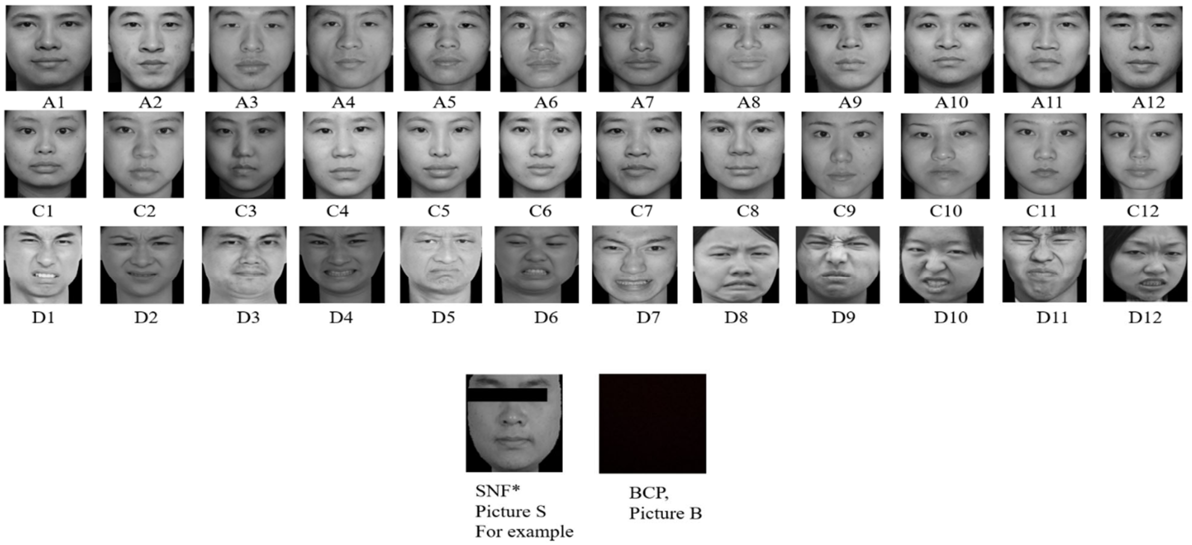

Figure S1. The images of faces. * To protect the subjects' privacy, the eyes of the self-face were covered in this example. Abbreviations: A, others neutral face-male; C, others neutral face-female; D, others disgust face, sex random.

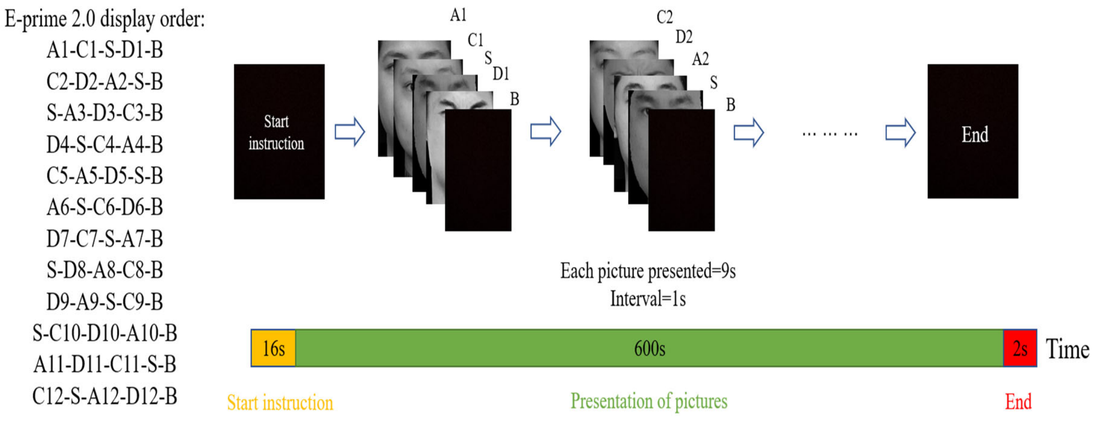

Figure S2. Stimulus design: Sequence of presentation during the task of self-face recognition via e-prime 2.0. Abbreviations: A, others neutral face-male; C, others neutral face-female; D, others disgust face, sex random.

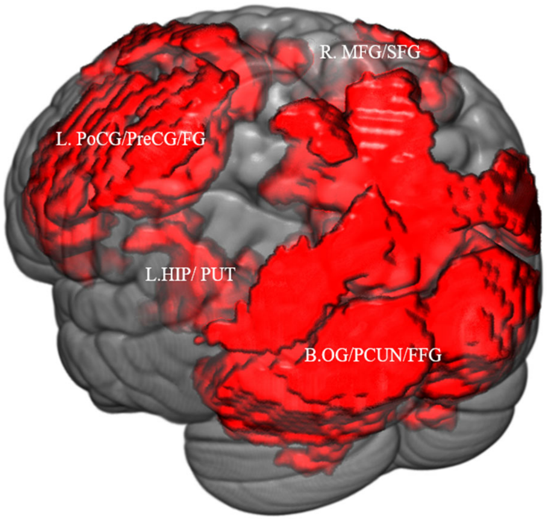

Figure S3. Results of two-way ANCOVA in the main effect of three conditions: (GRF correction: Voxel $p<0.001$, Cluster $p<0.05$, two tailed); condition 1: SNF vs BCP; condition 2: SNF vs ONF; condition 3: SNF vs ODF; Abbreviations: SNF, self-neutral face; ONF, others neutral face; ODF, others disgust face; R, right; L, left; PoCG, postcentral gyrus; PreCG, precentral gyrus; FG, prefrontal gyrus; MFG, middle prefrontal gyrus; SFG, superior prefrontal gyrus; HIP, hippocampus; PUT, putamen; OG, occipital gyrus; PCUN, precuneus; FFG, fusiform gyrus.

Table S2. Group difference of mean activation values between FEMDD and HC.

Brain areas	Values of the activation signal in FEMDD (Mean ± SD)	Values of the activation signal in HC (Mean ± SD)	*t* values	*p* values	Contrast
Significant interaction Effect					
Left FFG/IOG and Left CAL/ MOG/IOG					
Condition 1: SNF vs BCP	1.63±1.52	2.77±1.31	-3.73	0.00*	FEMDD＜HC
Condition 2: SNF vs ONF	0.07±0.41	0.24±0.30	-2.18	0.03*	FEMDD＜HC
Condition 3: SNF vs ODF	-0.04±0.39	0.02±0.40	-0.71	0.48	No significant
Simple effect of T test:					
Bilateral OG/FFG (Condition1: SNF vs BCP)	1.57±1.47	2.56±1.16	-3.41	0.00*	FEMDD＜HC
Right IFG/INS (Condition2: SNF vs ONF)	-0.07±0.21	0.08± 0.15	-3.67	0.00*	FEMDD＜HC

Note: *: $p < 0.05$; Abbreviations: SNF, self-neutral face; ONF, others neutral face; ODF, others disgust face; BCP, black control picture; FEMDD, first-episode major depressive disorder; HC, healthy control; FFG, fusiform gyrus; IOG, inferior occipital gyrus; CAL, calcarine; MOG, middle occipital gyrus; IOG, inferior occipital gyrus; IFG, inferior prefrontal gyrus; INS, insula.

Table S3. Pearson correlation between abnormal brain activation and SDS respectively in FEMDD and HC (p<0.05, two-tailed).

Brain areas	Values of the activation signal in FEMDD (Mean ± SD)	FEMDD-SDS	Values of the activation signal in HC (Mean ± SD)	HC-SDS
Bilateral occipital gyrus/fusiform gyrus Condition1: SNF vs BCP	1.57±1.47	r=0.19, p=0.14	2.56±1.16	r=0.04, p=0.83
Right inferior frontal gyrus/Insula Condition2: SNF vs ONF	-0.07±0.21	r=-0.10, p=0.43	0.08±0.15	r=0.06, p=0.74

Abbreviations: SNF, self-neutral face; ONF, others neutral face; ODF, others disgust face; FEMDD, first-episode major depressive disorder; HC, healthy control; SDS, self-disgust scale.

Table S4. Pearson correlation between abnormal brain activation and HAMD respectively in FEMDD and HC (p<0.05, two-tailed).

Brain areas	Values of the activation signal in FEMDD (Mean ± SD)	FEMDD-HAMD	Values of the activation signal in HC (Mean ± SD)	HC- HAMD
Bilateral occipital gyrus/fusiform gyrus Condition1: SNF vs BCP	1.57±1.47	r=0.05, p=0.72	2.56±1.16	r=0.01, p=0.97
Right inferior frontal gyrus/Insula Condition2: SNF vs ONF	-0.07±0.21	r=0.05, p=0.72	0.08±0.15	r=-0.05, p=0.77

Abbreviations: SNF, self-neutral face; ONF, others neutral face; ODF, others disgust face; FEMDD, first-episode major depressive disorder; HC, healthy control; HAMD, Hamilton depression rating scale.

Table S5. Two-way ANCOVA analyze with two groups (FEMDD vs HC) by four conditions (ONF vs BCP, SNF vs BCP, SNF vs ONF and SNF vs ODF) with correction level of Gaussian Random Field (voxel p<0.001, cluster p<0.05, two-tailed, clusters size≥15).

	**Brain areas**	**Voxels**	**Peak MNI coordinates (x, y, z)**	***F*/*t* value**
	**Main effect of the two diagnostic groups**			
Left	Fusiform gyrus/Inferior occipital gyrus	52	-42 -78 -18	17.62
Right	Inferior occipital gyrus	16	45 -75 -9	16.18
Left	Superior medial frontal gyrus	23	-6 60 18	16.77
Right	Inferior frontal gyrus	34	48 6 18	17.99
Right	Superior Frontal gyrus	20	15 48 21	13.99
	Interaction effect: two groups by four conditions			
Left	Fusiform gyrus/Inferior occipital gyrus	27	-42 -75 -18	6.82
Right	Middle temporal gyrus	15	54 -63 0	7.69
	T-test between FEMDD vs HC in ONF vs BCP			
Left	Inferior occipital gyrus/Middle occipital gyrus/ Fusiform gyrus	240	-24 -99 -9	-4.45
Right	Inferior occipital gyrus/Middle occipital gyrus/Lingual gyrus	30	24 -93 -9	-3.64
Left	Middle occipital gyrus	33	-42 -87 15	4.54
	T-test between FEMDD vs HC in SNF vs BCP			
Left	Inferior occipital gyrus/Calcarine/ Fusiform gyrus	260	-42 -75 -18	-4.48
Right	Inferior Occipital gyrus/Middle occipital gyrus	72	45 -78 -9	-4.15
Left	Superior medial frontal gyrus/Superior frontal gyrus	53	-6 60 18	-4.02
Right	Inferior frontal gyrus	33	48 9 18	-3.81
	T-test between FEMDD vs HC in SNF vs ONF			
Right	Inferior frontal gyrus /Insula	76	48 3 15	-4.00
Right	Inferior temporal gyrus/Middle temporal gyrus	69	51 -63 0	-4.51
Left	Middle frontal gyrus/Superior frontal gyrus	30	-21 48 15	-4.15

Abbreviations: SNF, self-neutral face; ONF, others neutral face; ODF, others disgust face; BCP, background control picture; FEMDD, first-episode major depressive disorder; HC, healthy control.
